# The Significance of Pets for Vulnerable Older Adults during the COVID-19 Pandemic: An Explorative Qualitative Study

**DOI:** 10.3390/ani12202752

**Published:** 2022-10-13

**Authors:** Peter W. A. Reniers, Ruslan Leontjevas, Ine J. N. Declercq, Marie-José Enders-Slegers, Debby L. Gerritsen, Karin Hediger

**Affiliations:** 1 Faculty of Psychology, Open University of the Netherlands, 6419 AT Heerlen, The Netherlands; 2 Department of Primary and Community Care, Radboud Institute for Health Sciences, Radboudumc Alzheimer Center, Radboud University Medical Center, 6500 HB Nijmegen, The Netherlands; 3 Faculty of Psychology, University of Basel, 4055 Basel, Switzerland

**Keywords:** older adults, pets, long-term care, community care, COVID-19

## Abstract

**Simple Summary:**

Pets may reduce the negative impact of COVID-19 countermeasures, such as social isolation in older adults receiving long-term care at home. To better understand the significance of pets for older adults in long-term care during the pandemic, we conducted interviews with five older adults in long-term care at home and with four family caregivers. All participants reported having negative experiences during COVID-19 countermeasures. The results suggest that pets were a source of structure and stability for pet owners. The study showed that pets played an important role in the lives of older adults in long-term care at home both before and during the pandemic.

**Abstract:**

Older adults receiving long-term care at home (LTCH-clients) were impacted by the COVID-19 pandemic and its countermeasures. Previous research suggests that pets can mitigate some of the pandemic’s impacts for older adults but results are contradictory. Our aim was to investigate experiences of LTCH-clients and the significance of their pets during the pandemic. Accounting for saturation, semi-structured interviews were conducted with five LTCH-clients and four family caregivers of LTCH-clients with dementia. Participants were asked about their experiences with COVID-19 and the significance of LTCH-clients’ pets during the pandemic. Two researchers performed thematic analyses in ATLAS.ti using open coding and an iterative–inductive approach. All participants reported negative experiences as a result of COVID-19 countermeasures. Results suggested that caring for pets provided pet owners with structure, which may have contributed to a sense of stability and continuity. Our outcomes underlined an important role of pets for LTCH-clients both before and during the pandemic.

## 1. Introduction

Older adults in general and older adults receiving long-term care at home (LTCH-clients) were impacted by the COVID-19 pandemic and its countermeasures [1,2,3]. For example, older adults were visited less frequently by friends and family because of measures imposing social distancing. This resulted in social isolation and loneliness in some older adults [4,5,6,7]. The risk of loneliness was already higher for older adults before the pandemic due to various cohort characteristics, such as less contact with former work colleagues after retirement, increased illness and disability, decreased mobility, and loss of friends, relatives, or partners. There is an increased risk for loneliness for those who live alone, have a small social network, or do not participate in social activities [8]. Social isolation and loneliness are risk factors for mental health problems, such as anxiety and depression, and for cardiovascular health problems, which can lead to higher mortality rates [9,10].

Pets are suggested to have been a source of support during the COVID-19 pandemic [11]. Approximately half of Western households own pets [12]. Many LTCH-clients own pets as well, although no numbers have been reported for this specific population. Recent studies have found that owning a pet during the pandemic could have beneficial as well as detrimental effects on the owners. The reported benefits of pet ownership included companionship, distraction, improved subjective wellbeing, and reduced loneliness and psychological distress [10,13,14,15]. Detrimental effects included increased worries about contracting COVID-19 from a pet, worries about a pet’s care if the owner were to become ill, and concerns about access to pet food, supplies, and services [13,14,16,17,18,19]. There is some evidence that pet owners had higher activity levels and better emotional wellbeing during the pandemic than non-pet owners and that this association became more apparent with increased age [20]. Another study found that pet ownership during the pandemic was associated with lower levels of wellbeing. However, when the researchers in this study accounted for demographics, this was especially true for women, households with two or more children, and people who were unemployed [21]. This indicates that demographics may also play a role in understanding the effects of pets during the pandemic. Therefore, pets may have supported LTCH-clients during the pandemic, but these owners may also have suffered some of the negative consequences of pet ownership.

A study conducted in Switzerland found that older adult pet owners more often lived in a house than in an apartment and that pet ownership decreased with age. Pet owners of 75 years and older, those living in an apartment, and those without a partner were more likely to benefit from owning pets. However, study participants also reported financial costs as a challenge, especially for those with lower levels of education and smaller budgets [22].

Currently, the significance of pet ownership for LTCH-clients during the pandemic is unknown as there have been no studies to date on this topic and previous research conducted in the general population cannot be generalised to LTCH-clients. Knowledge on pet ownership for LTHC-clients is highly relevant for improving support for this population—mainly due to potential benefits of pet ownership, such as reducing loneliness and distress. This topic is of interest due to ageing populations and health care reforms which prioritise long-term care at home over institutionalised care [23]. The aim of this study was to gain insight into (1) the influence of the pandemic and its countermeasures on the lives of LTCH-clients and (2) the significance of pets to LTCH-clients during the pandemic.

## 2. Materials and Methods

### 2.1. Study Design and Procedures

We recruited either LTCH-clients with pets or the family caregivers of LTCH-clients with pets when clients could not be interviewed themselves. Family caregivers may provide insight into the experiences of LTCH-clients with dementia by proxy. Reporting by proxy is a method that is used in vulnerable groups, such as people with dementia, who are not capable or limited in providing self-reports [24,25]. Family caregiver perspectives may also provide additional insight into the LTCH-context [26]. Participants were recruited using purposeful sampling through the networks of two Dutch community-care organisations operating in the southeast region of the Netherlands. Community care workers distributed an information letter about the study to pet-owning LTCH-clients. Prospective participants were able to sign up by phone or email or by giving permission to the community-care workers to be contacted by the research group. Participants could indicate whether they preferred to be interviewed by phone, over Microsoft Teams, or during a personal visit by a researcher. The recruitment procedure was guided by the data saturation principle [27].

A total of nine interviews were conducted, five with LTCH-clients and four with family caregivers of other LTCH-clients with dementia. Interviews took place between 18 May 2021, and 21 September 2021, and lasted between 16 and 65 min. Eight of the interviews were conducted by a PhD student (PR), and one of the interviews was conducted by a research assistant (ID).

Four interviews (two with LTCH-clients and two with family caregivers) were conducted by telephone; two interviews (with family caregivers) were conducted using Microsoft Teams; and three interviews were conducted in person at the LTCH-clients’ residences. Face-to-face interviews were recorded using a USB digital voice recorder (brand: LifeGoods). The interviews conducted over Microsoft Teams were recorded using a feature in the program and phone interviews were recorded using the Microsoft Windows 10 Voice Recorder software on a university laptop. The recordings were subsequently transcribed verbatim and pseudonymised.

Before the main interview started, all participants provided informed consent, either in writing or in an audio or video recording. The documentation of informed consent was saved and stored separately from the main interview in accordance with the study protocol that was approved by the ethics committee of the Open Universiteit (U202102010, U202103844). After transcription and analyses, the audio and video recordings of the main interviews were deleted. Criteria for reporting qualitative studies (COREQ) were considered while writing the manuscript [28].

### 2.2. Questionnaire

The semi-structured interview protocol was based on existing questionnaires and literature on experienced support and attachment to pets and social support [17,29,30]. Furthermore, some additional questions were formulated about participants’ experiences during the pandemic and quality of life [31]. The interview protocol was developed and refined through discussions with experts in anthrozoology (KH and ME) and psychogeriatrics (DG and RL). An example of a question we asked is: Can you tell us something about how the COVID-19 pandemic affected you? In addition to open-ended questions, the participants were asked to answer questions based on a scale from 1 to 10—for example: What score from 1 to 10 would you give the bond with your pet? For this question, a higher score indicated a better bond. The answers to these questions were used to describe sample characteristics. The full interview protocol can be found in Appendix A.

### 2.3. Analysis

We applied a thematic analysis using open coding and an iterative–inductive approach [32] in ATLAS.ti 9 for Windows (ATLAS.ti Scientific Software Development GmBH). Two researchers, P.W.A.R. and I.J.N.D., independently coded seven interviews. The two researchers compared and discussed their initial codes by using ATLAS.ti’s intercoder agreement function. Through comparison and inductive reasoning, the two researchers determined a first set of common themes from the interviews. The researchers subsequently refined these themes during a workgroup discussion with experts in anthrozoology and psychogeriatics (M.-J.E.-S., K.H. and R.L.) until consensus was reached. To determine data saturation, researchers conducted two additional interviews. No new themes emerged from these interviews.

During one of the interviews, it emerged that the interviewee, a family caregiver, cared for her mother who lived in a residential facility. Therefore, only part of the interview protocol was conducted with her. In another interview, it came to light that the participant often cared for the pets of people in her network but did not own a pet of her own. Yet, she claimed to be especially attached to a dog she often took care of. The researcher decided to continue interviewing her, since other research indicates that the effects of pets on people are often the result of attachment to a pet, rather than simply being its owner [33]. Although these two participants did not entirely fit our inclusion criteria, their answers were not excluded.

## 3. Results

### 3.1. Participant Characteristics

See Table 1 for participants characteristics. All participant interviews were related to dogs, except for one, in which case the LTCH-client owned a cat. CL5 regularly cared for the dogs of others, and IC4 cared for her mother who lived in a residential facility.

All participants indicated that they were highly attached to their pets. Participants were asked to rate the bond with their pet from 1 to 10, with 10 indicating a high degree of attachment. Ratings varied between 7 and 10. The score most often given was 10 (N = 3). Additionally, participants were asked to rate the support they received from their pet. Ratings varied between 7 (N = 4) and 10. When participants were asked how much they would miss their pet if it were no longer here, ratings varied between 7 and 10, with a score of 8 given most frequently (N = 5).

When asked how much the COVID-19 pandemic had impacted participants, the scores varied greatly. One LTCH-client and one family caregiver replied that COVID-19 had not impacted them at all (score of 1). Other participants’ scores varied between 6 and 8. One LTCH-client did not provide a rating.

Participants scored the quality and extent of support provided by family and friends between 7 and 10 (the most common score was 8, N = 4). The quality and amount of support provided by formal sources (e.g., from community care workers) was rated between 8 and 10 (most common was 9, N = 4). See Appendix B and Appendix C for all ratings and Table 2 for the themes retrieved from the interviews.

### 3.2. Daily Life-Related Themes

#### 3.2.1. Countermeasure Inconveniences

During interviews, participants reported experiencing inconveniences resulting from pandemic countermeasures, such as those related to face masks. For instance, face masks made other people more difficult to understand since the mask blocked or muffled the speaker’s voice during conversations. One participant said that wearing a mask caused her glasses to fog over, interfering with her ability to see. Another participant reported that he forgot to bring his mask on a trip to the supermarket, where wearing a mask was mandatory. Another participant felt inconvenienced by the closure of so many facilities, such as restaurants and bars. Participants also characterised restrictions on the number of visitors allowed in one’s home as a negative consequence of the pandemic. One participant indicated that he was skeptical of COVID-19 and the implemented countermeasures, calling it a charade.

CL3: “*When I arrived at the store… I had forgotten my face mask… So, I had to beg other people for a mask …*”.

#### 3.2.2. Relational Aspects

Some participants said that the COVID-19 pandemic had had a severe impact on their relationships and social networks. One participant reported having lost several family members due to COVID-19. Another reported that she still had visitors but that they maintained social distance.

A family caregiver reported that she vented her work-related stress at home on her husband with dementia. This made him sad or angry. She also reported that the COVID-19 countermeasures had made her husband’s illness less confronting. Social events were becoming increasingly difficult to partake in because of her husband’s stage of dementia but due to pandemic countermeasures she did not have to worry about such events.

CL4: “*Well not a lot of visitors… we… did keep our distance… Because my friend, she would still come inside sometimes… and the groceries, we had those delivered… I didn’t go out for groceries myself…*”.

#### 3.2.3. Work-Related Aspects

Some family caregivers spent more time at home due to work-from-home mandates and some reported experiencing additional work pressure due to the pandemic. Colleagues were out on sick leave more often during the pandemic than they were before the pandemic, which increased the workload for other colleagues. Additionally, some participants found employer-requested testing for COVID-19 (e.g., after contact with a colleague who had tested positive) stressful. These work-related aspects may indirectly impact the relationship with the LTCH-care recipients (see the example in Relational Aspects).

IC4: “*During the past period, I regularly had the problem that people were quarantined and couldn’t work… and me too… When one of the roommates, who had a cold… well then you have to get tested and well yeah… that always causes stress*”.

### 3.3. Pet-Related Themes

#### 3.3.1. Stability, Continuity

When asked whether the significance of their pets had changed for them during the pandemic, none of the participants reported any change. One family caregiver did indicate that she was at home more often than before, and that her dog forced her to go outside. Additionally, none of the participants reported additional worries concerning pet care during the pandemic. Participants experienced their pets and cared for them just as they had done before the pandemic. The indications were that pet ownership remained a stable factor that provided continuity and structure during the COVID-19 pandemic.

CL2: “*Well, I cannot say that it has changed… no… Because she has gotten everything she usually had, so… She went out once a day and good food and such… So, in that sense nothing changed… And inside… nothing really changed either*.”

#### 3.3.2. Attachment

Both LTCH-clients and family caregivers considered their pets to be reliable and trustworthy friends who provided emotional support, offered distraction, and reduced loneliness. Pets also provided participants with a sense of safety—for instance, by barking at potential danger.

CL5: “*Yeah, so uh… and it is a sweet animal and also a good guard animal… I would say if someone rings the doorbell then he barks… But, in the evening then he doesn’t bark… in the evenings, he is always on his bench so…*”.

#### 3.3.3. Activities

Pet-related chores, such as dog walking, continued throughout the pandemic and helped people maintain a certain level of activity. Pets were perceived as providing various health benefits to their owners.

CL1: “*That dog keeps me going also, that happens unnoticed… the walking and uh… get on the sidewalk… get off the sidewalk… and everything that you encounter on your way… but what if I didn’t have that dog anymore… what would you do? Read the newspaper?*”.

#### 3.3.4. Social Contact

Participants often had conversations with other people they met while walking their dog. Participants indicated that it was easier to connect to other dog owners, since they had a mutual interest. When it was permitted, some participants took their dogs along to visit family members. Additional social contact was not limited to dog owners as the cat owner had a friend that came over regularly to clean the cat’s litter box.

IC4: “*Anyway… it’s for your social contacts… Other dog owners they too uh… If you have mutual interests, it makes it easier to make contact and uh… yeah…*”.

Pets were considered a nice topic of conversation between people. A family caregiver mentioned that the pet was the only topic of conversation she had with her care recipient.

IC4: “*If someone goes there without the pet… Then it is difficult to connect… Difficult to communicate with her… And then you have uh… You have nothing to talk about*”.

#### 3.3.5. Pleasant Feelings

Participants viewed pets as a source of pleasant feelings and as contributors to a pleasant atmosphere. Pets gave a homely, cosy feeling to the house. Participants experienced their pets as being always enthusiastic and happy—for instance, when participants came home. This helped participants feel good about themselves.

IC1: “*He is always happy… yeah… you get support from it… from a dog… We always had dogs…*”.

#### 3.3.6. Downsides

Participants reported several downsides to pet ownership, none of which related to the pandemic. For example, pets were considered a lot of work, and sometimes they ruined possessions (e.g., a puppy that chewed on shoes). Some participants mentioned the difficulty of being away from home for long periods of time and having to walk the dog even in bad weather. One participant who owned older dogs indicated that she felt concerned about the dog’s upcoming death. The LTCH-client who owned a cat reported that his cat woke him up early in the morning, which he found undesirable.

CL3: “*Yeah yeah... and sometimes very persistently… There has been a time because I sleep downstairs out of necessity. That has also been taken care of through medical support by the way… that I have that bed… But uh… it is… yeah... because… Sometimes in the middle of the night she starts to hunt… and runs up and down the sofa over there that she has already ruined… and at five o’clock in the morning or something like that…*”.

## 4. Discussion

### 4.1. Findings

In this study, we identified three themes related to the daily life of participants that describe the impact of the pandemic and its countermeasures on LTCH-clients and their family caregivers—namely, countermeasure inconveniences, relational aspects, and work-related aspects. We also identified the following six themes concerning the roles of pets during the pandemic: stability or continuity, attachment, activities, social contact, pleasant feelings, and the downsides of caring for a pet. According to the participants, there were no significant changes in terms of the role of their pets played in these domains during the pandemic.

All of the participants reported experiencing some degree of inconvenience during the pandemic. Participants reported not being allowed to receive many visitors and finding this restriction undesirable. Such limitations on contact with others could potentially impact significant relationships. A longitudinal study conducted in the Netherlands a year prior to the current study showed that during the pandemic social and emotional loneliness increased in older adults, and that older adults had less social contact during this time. However, despite this finding, the mental health of participants was not impacted [34].

Work-related aspects were especially relevant for some family caregivers. For instance, due to pandemic countermeasures, family caregivers worked from home more often. A Canadian study showed that working from home may have a positive influence on the caregiving experience. Caregivers may have had more flexibility in their schedules, making it easier to organise care. On the other hand, caregivers may also have spent more time on caregiving tasks and, due to pandemic countermeasures, less formal caregiving may have been carried out. This may have had a negative effect on the caregiver burden [35].

The most surprising finding regarding pets in our study was that none of the LTCH-clients or family caregivers reported any change in the significance of their pets during the pandemic. Our study could not confirm the outcomes of studies in which relatively large groups of people reported a change in the relationship with their pets during the pandemic e.g., [17,19]. For instance, in a study conducted in Spain (N = 1297) almost 30% of dog owners and 52% of cat owners indicated an improved relationship with their pet, and 6% of dog owners and 1.5% of cat owners reported a worse relationship [17]. These results show that previous findings in general populations cannot simply be generalised to LTCH-clients. The topic needs further in-depth investigation for this specific group in the future.

In our study, the participants reported no additional worries or concerns during the pandemic regarding their pets’ care. This is contrary to other studies that reported additional worries related to pet care, e.g., [16,18]. However, a more recent publication including 5454 pet owners from various countries also showed that most pet owners did not report additional concerns related to their pets’ care during the pandemic [36]. More research on this subject is recommended.

There are several possible explanations as to why the participants in our study reported no changes in the pets’ significance or additional worries concerning the pet. First, corresponding to the themes activities and stability, continuity, retired older adults experienced fewer changes in their lives due to the pandemic than employed younger adults did [37]. Specifically, LTCH-clients were already more homebound pre-pandemic than the general population. Nevertheless, the countermeasures, such as the limitation on visitors and closed facilities, e.g., restaurants, where they could meet others made the already small world of LTCH-clients even smaller. This might have impacted their quality of life, and this may explain the scores some participants gave when they were asked how much they were burdened by COVID-19 (Appendix B). In spite of that, LTCH-clients could still perform the same activities with their pets during the pandemic as they did before the pandemic, and the significance of their pets did not change during this time. This suggests that pets provided continuity and stability that made it easier to adapt to a world with COVID-19 and its countermeasures and helped to maintain their quality of life.

A second explanation, which corresponds to the themes attachment and pleasant feelings, is that research has shown that attachment to a pet is a protective factor when it comes to exacerbating mental health symptoms [38]. The participants in our study indicated feeling a high degree of attachment to their pets and said that their pets helped them experience pleasant feelings. The presence of pets may have protected LTCH-clients against some of the negative effects of COVID-19 and its countermeasures. However, the results of a study that investigated adults in the UK showed that stronger attachment to a pet was related to higher reported rates of depression and loneliness and a lower degree of self-perceived positive experiences during a period of lockdown [39]. The results of a Brazilian study, on the other hand, are in line with our study. The Brazilian study showed that dog ownership—as opposed to ownership of any other type of animal—and physical activity were associated with less depression during lockdown [40].

Third, all but one of the participants in our study cared for a dog(s). Dog ownership rather than cat ownership has been associated with self-reported successful ageing, better functional ability, and fewer chronic illnesses in older adults who experience low levels of social support [41]. In a survey of 767 dog owners and 767 potential dog owners conducted in the United States during the pandemic, dog owners scored higher on levels of social support and had slightly lower depression scores than potential dog owners. The results from that study seem to indicate that the effect on depression can be explained mainly by the higher level of social support experienced by dog owners. Yet, dog ownership may have facilitated or provided an extra sense of social support in the group of dog owners [42]. This outcome is supported by an Australian study that found that as opposed to cat owners, dog owners were less likely to experience loneliness during lockdown. The authors provided an explanation based on qualitative responses of their participants in which they indicated that dog owners had to go outside to walk their dog, which provided structure (related to the theme stability, continuity) and enabled them to meet other people (related to the theme social contacts) who were also walking their dog [10].

The examples above indicate that pet ownership—and especially dog ownership—is beneficial during times of social isolation and may be especially helpful for older adults who experience low levels of social support. Some studies that found a reduction in older adults’ quality of life during the pandemic attributed this to decreased social contact and decreased help with functional needs, such as help with washing [37]. The participants in our study indicated that they met other people while walking the dog. Therefore, the pet facilitated additional social contact for the participants in our study. Contradictory to this finding is a longitudinal quantitative study performed between 2018 and the first Australian lockdown in 2020 in a sample of LTCH-clients (N = 21) that found a significant decrease in the quality of life of LTCH-clients during the pandemic; however, the study found no changes in the LTCH-clients’ networks of family and friends during lockdown and could not confirm a relationship between the social network and the experienced quality of life [43].

Fourth, in our study, all participants rated support from formal and informal sources as sufficient and high quality. This result may indicate that the participants’ social networks remained stable and supportive during the pandemic. A person’s experience of the significance of a pet may be related to the amount of social support a person receives. For instance, a survey study with 830 older adult primary-care clients has shown that living alone is the best predictor of loneliness in older adults. Yet, when older adults living alone owned a pet, they were less likely to report loneliness [44]. This suggests that pets may be especially beneficial for older adults living alone.

The fifth and concluding reason is that older adults seem to be more resilient and better able to cope with stress and emotions than younger adults are [37]. This suggests not only that LTCH-clients may have experienced fewer changes during the pandemic but also that it is easier for them to mitigate these changes and the possible negative outcomes thereof themselves. Nevertheless, when asked about the possible downsides of owning a pet, most of the participants were able to provide some examples; however, none of the examples provided were connected to the pandemic (theme: downsides). Yet, the downside of not being able to stay away from home for too long is worth mentioning. There is evidence that pet owners delay COVID-19 testing or treatment due to concerns about a pet’s welfare, especially if they have no plan in place for a pet’s care [45]. This may pose an increased risk for older people and for those in poor health, as is the case with many LTCH-clients.

### 4.2. Limitations, Strengths and Future Research

A limitation of our study was that most of our participants cared for dogs only. Therefore, it is not possible to generalise the results to owners of other types of animals. Another limitation may be that we only interviewed a small sample. However, we used data saturation as a guiding principle. Sample sizes in qualitative studies are not guided by expected generalizability of statistical findings while meta-themes in qualitative data can be found in small groups of participants [27]. Further, the respondents were all from the same region in the Netherlands, and when we conducted the interviews, there were no hard lockdown measures in place [46]. It is therefore possible that the outcomes of our study were biased by the memory of the participants. For these reasons, caution is advised when generalising our findings to owners of other types of pets in other regions and countries, especially since COVID-19 countermeasures varied across regions, countries, and time.

To the best of our knowledge, there have been no previous studies that have specifically investigated the impact of the pandemic and the significance of pets therein within the LTCH-context. A strength of our study is that we looked at the subjective experiences of either LTCH-clients or the family caregivers of LTCH-clients with dementia. Quantitative (cross-sectional or longitudinal) research with validated instruments conducted in different populations with various types of pets could provide more clarity on the effects of pets in specific target groups. Future (quantitative) research could also help to clarify whether variables, such as the level of attachment, activity level, and positive affect, influence the effects of pets within the LTCH-context.

## 5. Conclusions

Our study suggests that the way LTCH-clients experienced and cared for their pets remained unchanged throughout the various stages of the pandemic. This implies that pets continually play an important role for LTCH-clients and may have helped maintain structure and stability during the COVID-19 pandemic. In addition to feeling attached to their pets, participants mentioned several positive aspects of their pets that were unrelated to the pandemic. In addition to providing stability, pets play a significant role in participants’ lives in terms of activities, social contact, and pleasant feelings. Although participants also identified some downsides of pet ownership, such as additional cleaning and having to walk the dog in bad weather, our results imply that pets were beneficial for LTCH-clients during the pandemic and that the pets’ wellbeing was not negatively affected.

## Figures and Tables

**Table 1:** Participant and Pet Characteristics.

Older Adults in LTC	Gender (Care Recipient)	Age (Care Recipient)	Pet (Breed)	Pet Age
CL1	Male	79	Dog (Labrador Retriever)	2.5
CL2	Male	80	Dog (Mixed Breed)	6
CL3	Male	82	Cat (Mixed Breed)	1
CL4	Female	66	Dog (Boomer)	9
CL5	Female	79	Dog (Mixed Breed)	2
Caregivers				
IC1	Male (Female)	71(64)	Dog (Boomer)	4
IC2	Female (Male)	65 (63)	Dog (Stabyhoun)	5
IC3	Female (Male)	65 (63)	Dogs (Jack Russel Terrier and Maltese Dog)	11, 11
IC4	Female (Female)	62 (unknown)	Dogs (Poodles)	14, 11, and 2.5

Note: Older adults in long-term care (CL) and family caregivers (IC) were not related.

**Table 2:** Themes and Example Codes retrieved from interviews about the pandemic and the role of pets.

Daily Life-Related Themes	Example Codes
Countermeasure Inconveniences	Face Masks Closed Facilities
Relational Aspects	Irritability Less Contact with Others
Work-Related Aspects	Working from Home Work Pressure
**Pet-Related Themes**	**Example Codes**
Stability, Continuity	Daily Care for the Pet Reliable
Attachment	Friendship Sense of Safety Emotional Support
Activities	Walking/Cycling Dog Goes on Outings with Owners
Social Contact	Meet New/Other People The Pet and Third Parties
Pleasant Feelings	Cosiness, Homely The Pet Greets Enthusiastically
Downsides	Cannot Go Out Too Long Dog Walking in Bad Weather

## Data Availability

Data is available on request peter.reniers@ou.nl.

## Appendix A

Questionnaire

Demographics

- What is your sex?
- What is your age?
- Are you a client or informal caregiver?

Main Interview

- What kind of pet do you own? Do you have more than one pet? What is the breed, age and health of the animal?
- How long have you owned your pet?
- What score would you give the bond with your pet from 1 to 10?
- What score would you give the support that you receive from your pet from 1 to 10?
- How much would you miss your pet if it would be gone from 1 to 10?
- What score would you give your current life?
- How much did the COVID-19 pandemic impact you from 1 to 10?
- Can you tell us something about how the COVID-19 pandemic affected you?
- What type of activities do you undertake with your pet?
- What does your pet mean to you? Can you provide some examples?
- Did the significance of your pet change during the pandemic? If so, how?
- Can you tell us something about downsides to owning a pet? Were there additional downsides due to the pandemic?
- Your pet has to be cared for. Do you rely on others to help you care for your pet? How?
- If others are partly responsible for your pet’s care, what does that mean to you? And for your relationship with this person?
- Do you have a partner? What does your household look like?
- How many friends and acquintances do you see regularly? Would you like to see them more or less often?
- Are you supported by friends and family? Could you use more support? If so, how?
- If you would have to give the amount and quality of support from friends and family a score from 1 to 10?
- How often do you see professional caregivers? Who are these?
- How do professional caregivers support you? Do you think this is sufficient?
- How would you score the amount and quality of support that your receive from professional caregivers from 1 to 10?
- This question is about the extent to which you are able to walk. A score of 1 means that you are unable to walk yourself and a score of 10 means that you experience no problems walking.
- This question is about the extent to which you are able to wash and dress yourself. A score of 1 means that you are unable to wash or dress yourself and 10 means that you have no problem washing or dressing yourself.
- This question is about the extent to which you can perform your activities of daily living (for instance, household, family, and leisure time activities). A score of 1 means that you are unable to perform your activities of daily living and 10 means you have no problems performing your activities of daily living.
- This question is about the extent to which you experience pain or discomfort. A score of 1 means that you experience extreme pain or discomfort and a score of 10 means you experience no pain or discomfort.
- This question is about your mood. A score of 1 means that you feel extremely anxious or sad and a score of 10 means you are not anxious or sad.
- Now we want to know how good or bad your health is TODAY on a scale from 1 to 100, with 100 being the best health you can imagine and 1 being the worst health that you can imagine.

## Appendix B

Participants’ Ratings on Pets, Life, COVID-19 Burden, and Social Support.

	**Attachment to Pet Rating**	**Support from Pet Rating**	**Missing the Pet Rating**	**Current Life Rating**	**Burdened by COVID-19 Rating**	**Support from Friends and Family**	**Support from Formal Caregivers Rating**
CL1	8	8	8	8	8	8	8
CL2	7 or 8	7	7	6 or 7	N/A	7	8 or 9
CL3	8	7	8	N/A	7	8	9
CL4	10	10	10	8 or 9	1	10	10
CL5	10	9	8	8 or 9	7 or 8	8	9
IC1 (Proxy)	8 (8)	8 (8)	10	6	7	7	10
IC2 (Proxy)	8 (varies daily)	7 (9)	8 (9)	5 (6 or 7)	6 (6)	7	9
IC3 (Proxy)	8 or 9 (10)	7 (8)	8 (10)	6 (8)	1 (1)	8	10
IC4 (Proxy)	8 (8)	8 (7)	8 (9)	8 (5)	6.5	N/A	N/A

Note. In the (proxy), family caregivers provided an estimation by proxy of their care recipients’ ratings where possible.

## Appendix C

Ratings on Quality of Life of Clients ^1^.

	**Ability to Walk**	**Ability to Dress/Wash**	**Ability to Perform ADL**	**Pain or Discomfort ^3^**	**Mood**	**Health Today**
CL1	7	7 or 8	8	10	7 or 8	70
CL2	6	5	6	7	7	65
CL3	6	7	6	6	7	75
CL4	5	3 or 4	3	5	10	50
CL5	N/A	10	10	Regularly	8	90
IC1 Proxy ^2^	7	1	1	10	7	50
IC2 Proxy ^2^	10	2	5	10	8	30
IC3 Proxy ^2^	5 or 6	1	8	7	10	80
IC4 Proxy ^2^	N/A	N/A	N/A	N/A	N/A	N/A

Note. ^1^. Questions were based on the EQ-5D, and were rated using a 10-point scale; ^2^. Proxy scores reflect family caregivers’ opinions on care recipients’ outcomes; ^3^. In the Pain and Discomfort column higher scores indicate less pain/better functioning.
